# Post-cholecystectomy Mirizzi’s syndrome: Magnetic Resonance Cholangiopancreatography Demonstration

**DOI:** 10.4103/1319-3767.70620

**Published:** 2010-10

**Authors:** Nisar A. Wani, Naseer A. Khan, Asif I. Shah, Abdul Q. Khan

**Affiliations:** Departments of Radiodiagnosis and Imaging India; 1 Gastroenterology India; 2 General Surgery, Sher-I-Kashmir Institute of Medical Sciences (SKIMS), Srinagar, Jammu & Kashmir, India

**Keywords:** Cystic duct remnant, ERCP, Mirizzi’s syndrome, MRCP, postcholecystectomy Mirizzi’s syndrome

## Abstract

A long cystic duct remnant may be found after laparoscopic cholecystectomy. Stone may form in the remnant cystic duct and can cause postcholecystectomy syndrome. Remnant cystic duct calculus may rarely result in postcholecystectomy Mirizzi’s syndrome. Traditionally, Mirizzi’s syndrome has been diagnosed with endoscopic retrograde cholangiopancreatography (ERCP) and treated with open surgery. We report a case of postcholecystectomy Mirizzi’s syndrome that developed 3 years after laparoscopic cholecystectomy. A non-invasive diagnosis of Mirizzi’s syndrome was made comprehensively by magnetic resonance cholangiopancreatography. Endoscopic stone removal was achieved successfully with ERCP without any complication.

Mirizzi’s syndrome results from the impaction of a stone in the cystic duct or gall bladder (GB) that impinges on the common hepatic duct (CHD) leading to mechanical obstruction by the stone itself or by secondary inflammation. Jaundice and recurrent cholangitis are two main presentations.[1] Mirizzi’s syndrome may occur after cholecystectomy due to stone formation in the remnant cystic duct. Preoperative diagnosis of Mirizzi’s syndrome is somewhat difficult and can be accomplished non-invasively with ultrasonography or MRCP, the latter being better of the two at delineating the bile duct anatomy.[1,2] Direct cholangiography using percutaneous transhepatic cholangiography (PTC) and ERCP can facilitate the preoperative diagnosis of Mirizzi’s syndrome and delineate the biliobiliary fistula, if present.[3] Traditionally, treatment of the Mirizzi’s syndrome has been surgical; the type of the procedure depending upon the type of Mirizzi’s syndrome.[1] In recent years, endoscopic therapy has been used more and more frequently in the therapy of Mirizzi’s syndrome. ERCP has been used both as an adjunct to traditional surgical therapy and as a primary therapy in patients who are thought to be high risk surgical candidates. We describe a case of post-cholecystectomy Mirizzi’s syndrome in a 33-year-old woman who was diagnosed with MRCP and successfully treated, primarily with ERCP.

## CASE REPORT

**Figure 1:**
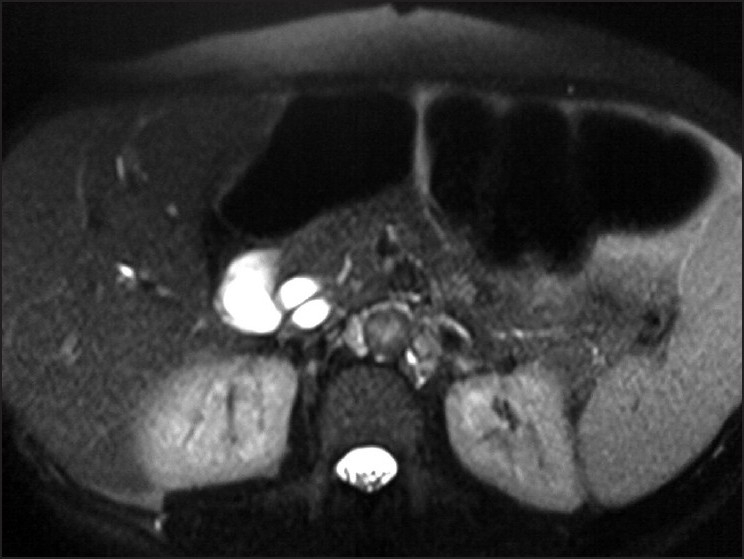
Axial heavily T2-weighted MR (MRCP) image showing closely related, parallel running common hepatic (CHD) duct (anterior) and cystic duct (posterior) in the region of pancreatic head

**Figure 2:**
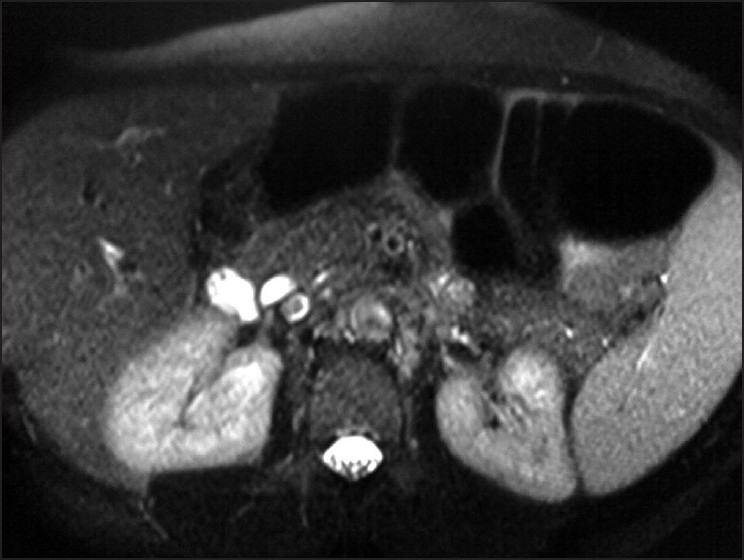
Axial MRCP image showing a filling defect in the posterior duct (cystic duct). Pancreatic duct is normal in caliber

**Figure 3:**
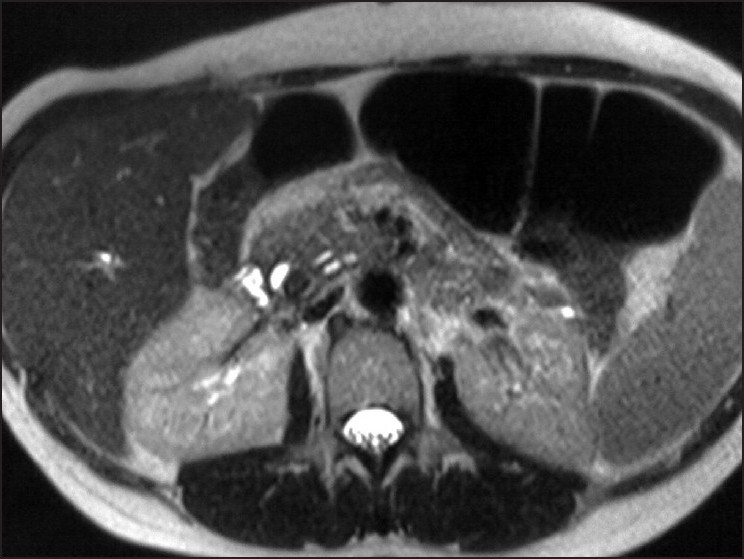
Axial MRCP image showing signal void filling the lumen of the posteriorly positioned cystic duct, and causing extrinsic compression of the anterior duct (CHD)

**Figure 4:**
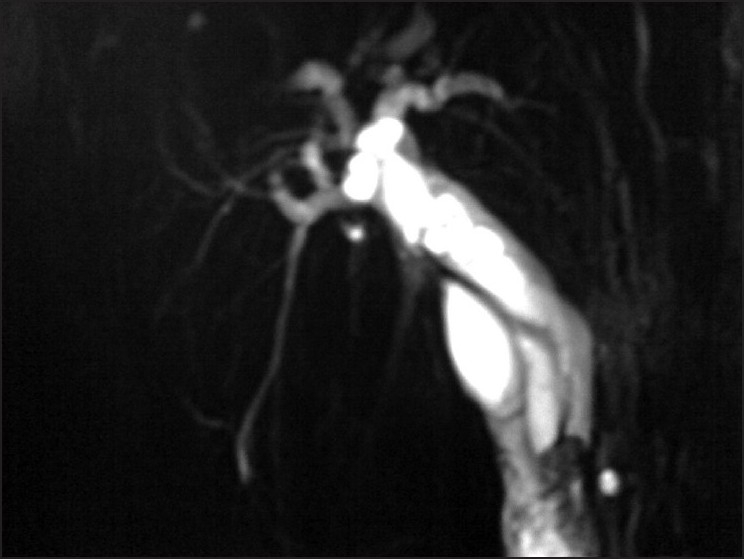
Thick slab oblique coronal plane MRCP image showing dilated intrahepatic bile ducts. Two overlapping and parallel running ducts (cystic duct and CHD) are seen with a signal void within cystic duct causing compression and obstruction of the common hepatic duct from the exterior. The common bile duct distal to obstruction is normal in diameter

**Figure 5:**
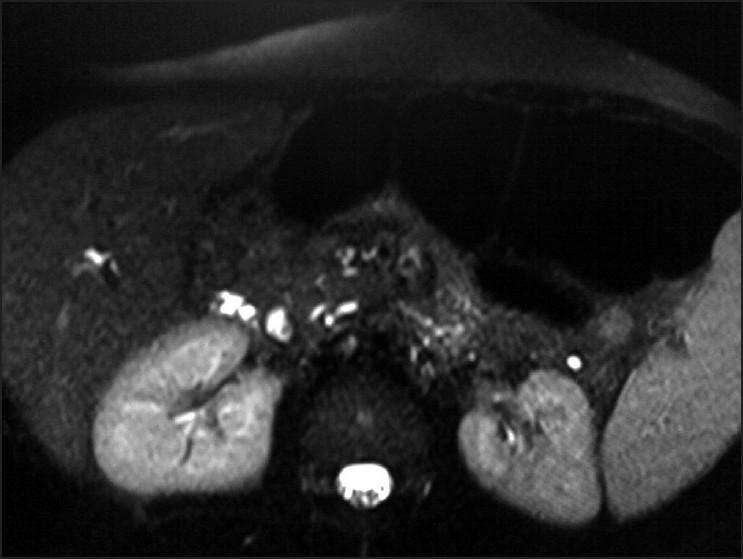
Axial MRCP image showing posteromedial insertion of the cystic duct into the distal portion of the common duct, which is of normal caliber compared to the dilated upper duct

A 33-year-old woman presented with complaints of upper abdominal pain, nausea, and yellowish discoloration of eyes from the last 30 days. There was no history of fever. She had undergone laparoscopic cholecystectomy for the symptomatic gall stone disease 3 years before. Right upper quadrant pain recurred 2.5 years after the surgery, for which the patient was treated empirically with H2 antagonists. Four weeks prior to admission, the abdominal pain became more severe and frequent, and radiated to her back. Empiric therapy with proton pump inhibitors and antacids yielded no response this time. On physical examination, the pulse rate was found to be 86 beats/min, temperature was 37.5° C, and blood pressure was 120/70 mmHg. There was evidence of scleral icterus. Abdominal examination revealed significant right upper quadrant tenderness; bowel sounds were normal. There were no stigmata of chronic liver disease. Laboratory studies revealed: alkaline phosphatase 840 U/L (70-320), AST 70 U/L (10-40), ALT 76 U/L (0-45), total bilirubin 4.5 mg/dL (0.2-1.5), direct bilirubin 2.1 mg/dL (0-0.5), serum albumin 4 g/dL (3.5-5.5) white blood cells 9× 10^9^/L (3.5-10.5), hemoglobin 13 gm/dL (12.0-15), polymorphonuclear neutrophils 6.5 × 10^9^/L (1.5-7.5), and prothrombin time of 14 s. In view of raised bilirubin with altered liver function tests (LFT) showing disproportional increase of ALP as compared to ALT/AST, ultrasonography (US) of the hepatobiliary system was performed to look for the cause of obstructive jaundice. On US, intrahepatic bile ducts (IHBDs) were dilated with dilatation of extrahepatic duct system upto its distal portion in the region of pancreatic head. A probable calculus was seen in relation to the distal common bile duct. MRCP was performed for the further characterization of the extrahepatic biliary tract anatomy. It showed a long cystic duct remnant running parallel [Figures 1 and 2] to the common duct with a 1.5 cm calculus within the terminal portion of the cystic duct [Figures 2–4] just above its insertion into the posteromedial aspect of the common duct [Figure 5] in the region of pancreatic head. The cystic duct calculus caused extrinsic compression [Figure 3] of the common duct with dilated upper duct and IHBDs [Figure 4] with normal caliber of the common bile duct distal to the site of obstruction [Figures 4 and 5]. The findings were compatible with a long remnant cystic duct with remnant duct calculus causing extrinsic compression and obstruction of common duct i.e. postcholecystectomy Mirizzi’s syndrome. We could not ascertain definitely whether it was a case of residual or recurrent remnant cystic duct calculus, as no preoperative cholangiogram was done. However, in view of a few years of asymptomatic period after cholecystectomy, we presumed recurrence as the cause of remnant cystic duct stone. ERCP was performed for a possible endoscopic therapeutic procedure. It confirmed the MRCP finding of extrinsic compression of distal common duct due to a calculus in the terminal portion of long remnant cystic duct; there was no definite evidence of biliobiliary fistula. Distal common bile duct was entered easily after pappilotomy; access into the proximal common hepatic duct and cystic ducts was achieved. Cystic duct stone was initially broken down with a mechanical lithotripsy basket that was advanced into the proximal portion of the cystic duct. Stone fragments were removed from the cystic duct with a balloon. Repeat ERCP showed patent common hepatic and bile ducts with a long remnant cystic duct. There was no post-procedure cystic duct leak. Patient’s jaundice disappeared and her LFT started to return to normal. She was discharged on third day after the procedure and is currently under follow-up.

## DISCUSSION

The underlying cause of postcholecystectomy syndrome (PCS) may be biliary stricture, retained or recurrent biliary calculi, stenosis or dyskinesia of Sphinter of Oddi, remnant gall bladder/cystic duct stump calculi, etc. Occluding stones left in the stump of cystic duct may account for 17-25% of cases of PCS.[2] Cystic duct remnant defined as residual duct greater than 1 cm in length may in presence of stones cause post-cholecystectomy syndrome. Remnant cystic duct calculus could either be due to the retained stone during the cholecystectomy or recurrence of stone in the actual remnant of the cystic duct.[2] A long cystic duct, which predisposes to the postcholecystectomy stone formation has a long course parallel to the common duct, and may have a low median insertion into the terminal common duct.[3] Stone within such a duct may cause common duct obstruction by extrinsic compression called Mirizzi’s syndrome.

Mirizzi first described this phenomenon as “functional hepatic syndrome” in 1948. Mc Sherry *et al*. in 1982 suggested a sub-classification of Mirizzi’s syndrome into two types; Csendes *et al*. 1989 described four types of Mirizzi’s syndrome.[4,5] The modern definition of Mirizzi’s syndrome is thought to include four components: impaction of stone in cystic duct or neck of gallbladder; mechanical obstruction of CHD by the stone itself or secondary inflammation; intermittent or constant jaundice causing possible recurrent cholangitis, and if long standing secondary biliary cirrhosis. Biliobiliary fistula may or may not be present.[1]

The diagnosis of Mirizzi’s syndrome may be suggested at imaging, when a stone is identified at the junction of the cystic duct and extrahepatic bile duct and is seen in conjunction with dilatation of the bile ducts proximal to the stone and a normal-caliber duct distal to the stone.[3] The sonographic findings of intrahepatic biliary dilatation, a normal-caliber common bile duct, and a large stone in the neck of the gallbladder or cystic duct suggest the diagnosis of Mirizzi’s syndrome. CT may reveal an abrupt narrowing of the suprapancreatic common duct in the absence of a mass lesion, suggesting Mirizzi’s syndrome. An important role of ultrasound or CT is to exclude a mass in the porta hepatis as a cause of extrahepatic obstruction. Though both CT and US demonstrate bile duct dilation proximal to the site of obstruction in Mirizzi’s syndrome, stones may not be apparent. The inflammatory reaction around the obstructing stone may mimic malignant obstruction.[6] Invasive cholangiography through the percutaneous transhepatic route (PTC) or endoscopic route (ERCP) has been essential to confirm the diagnosis of Mirizzi’s syndrome and to determine whether a fistula is present. ERCP is thought to be superior to PTC in this setting for several reasons.[7] Cystic duct may run parallel to the common hepatic duct and join it far below the usual meeting point. The anomalous insertion and long parallel course of the cystic duct cannot be well visualized by PTC but is easily recognized by ERCP. Another potential problem with PTC is that with significant obstruction of the common hepatic duct, the distal common bile duct may not be adequately visualized, ERCP may circumvent this problem.[7]

MRCP is the optimal method for evaluating biliary tree in the patients of postcholecystectomy syndrome with cystic duct remnant, as it is non-invasive.[2] MRCP can demonstrate the cystic duct stone causing extrinsic compression of the common hepatic duct at the junction of the two, besides demonstrating the proximal dilated duct system and normal caliber common bile duct distal to the site of obstruction.[3] Because of the close parallel course of the cystic duct and common duct that predisposes to the Mirizzi’s syndrome, overlapping of the two ducts may result in the thick slab projectional MRCP images. However, thin slice source MRCP images and heavily T2-weighted axial MR images can easily demonstrate the closely lying cystic and common ducts. Cystic duct stone causing extrinsic compression of the common duct can be very well seen in the axial MRCP images. Low inserting cystic duct may be mistaken for the common duct on ERCP resulting in inadvertent placement of the stent or stone extraction basket or balloon into the adjacent duct than the intended one^3^; this uncertainty and possible complications can be avoided by performing MRCP prior to ERCP. Thus, MRCP may provide a noninvasive alternative to ERCP and PTC in the diagnosis of Mirizzi’s syndrome.[8]

Surgery was the only standard therapy for Mirizzi’s syndrome some 15-20 years back. In the last two decades, there are several case reports and series that describe endoscopic and percutaneous adjunctive and alternatives to open surgery. These techniques include palliative decompression of the obstructed biliary system by the nasobiliary catheter or by internal endoprosthesis, using ERCP, to tide over acute infection or in poor surgical risk patients. Other non-operative alternatives include stone manipulation and disimpaction back to the gallbladder, percutaneous lithotripsy, endoscopic electrohydraulic lithotripsy, extracorporeal shockwave lithotripsy, and chemical dissolution.[6,7,9,10] Binmoeller *et al*. successfully treated largest series of patients with Mirizzi’s syndrome using electrohydraulic lithotripsy. The only complication was a post-procedural cystic duct leak, which was successfully managed nonoperatively.[9] Postcholecystectomy cystic duct remnant calculi were formerly managed with surgery, preferably laparoscopically. During the last two decades, cases have been reported in which cystic duct remnant stones were treated endoscopically.[10] Kodali and Petersen described two patients with postcholecystectomy Mirizzi’s syndrome that were treated by ERCP using standard balloon and mechanical lithotripsy approaches.[6] Access to the cystic duct above the stone may be most easily achieved with the assistance of a slippery hydrophilic guide wire. Forceful withdrawal of an intact stone from the cystic duct certainly carries some risk for duct perforation. Such small post-procedure leaks are likely to close with endoscopic management, just as postoperative leaks do.[6,7] Our case exemplifies the usefulness of MRCP for non-invasive diagnosis and effectiveness of the primary endoscopic removal of remnant cystic duct calculi causing Mirizzi’s syndrome, without any significant complication.

We conclude that cystic duct remnant calculus with low insertion of the cystic duct into the distal common duct may predispose to postcholecystectomy Mirizzi’s syndrome. This can be non-invasively diagnosed with axial MRCP images, thus providing roadmap for the therapeutic ERCP. Endoscopic therapy is an effective and safe alternative to open surgery for postcholecystectomy Mirizzi’s syndrome.
